# The Brief Case: Pneumonia in a *Boa constrictor occidentalis* with inclusion body disease caused by multidrug-resistant VIM-type metallo-β-lactamase producing *Pseudomonas aeruginosa*

**DOI:** 10.1128/jcm.01345-25

**Published:** 2026-03-11

**Authors:** Anthony Broering Ferreira, Ricardo Antonio Pilegi Sfaciotte, Lara Duque Estrada Meyer Fagundes, Heloíse Peterle, Roberta Farias Veiga, Sally Vieira, Rafael Kretzer Carneiro, Ubirajara Maciel da Costa, Sandra Maria Ferraz

**Affiliations:** 1 CEDIMA, Centro de Diagnóstico Microbiológico Animal, Department of Veterinary Medicine, Santa Catarina State University 357957 , Lages, Santa Catarina, Brazil; 2 Department of Veterinary Science, Federal Rural University of Rio de Janeiro 28125, Rio de Janeiro, Brazil; University of California, Davis, California, USA

**Keywords:** antimicrobial resistant, snake, metallobeta-lactamase

## CASE

**Fig 1:**
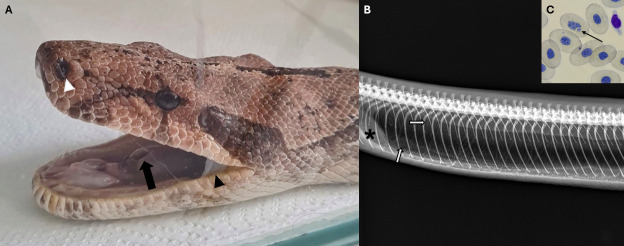
Seven-year-old female *Boa constrictor occidentalis* with mucous discharge from the oral and nasal cavities and oral breathing. (**A**) White arrowhead: nostril with dried secretion; black arrow: open glottis during oral breathing; black arrowhead: mucous secretion. (**B**) Radiograph of the pulmonary region in the left lateral position. Black asterisk: heart; white arrow: faveolar pattern, consistent with pneumonia. (**C**) Black arrow: inclusion body in erythrocyte.

A 7-year-old, 6.5 kg female Argentine *Boa constrictor* (*Boa constrictor occidentalis*) was presented with mucous discharge from the oral and nasal cavities, dyspnea, orthopneic posture, oral breathing, and respiratory crackles. The animal had a previous diagnosis of Boid Inclusion Body Disease (BIBD) confirmed by cytological examination, as well as a history of two previous episodes of pneumonia in recent years, and was submitted to radiographic examination (Fig. 1). Based on clinical history, clinical signs, and radiographic findings, a diagnosis of pneumonia was established.

The first episode of pneumonia occurred 1 year prior to the current presentation and was treated with 14 intramuscular applications of amikacin (5 mg/kg, q72h), associated with intracoelomic administration of 0.9% saline solution at 1% of body weight. The second episode occurred 7 months before the present case, with treatment initially consisting of amikacin and 0.9% saline solution; however, after four applications, no clinical improvement was observed. The therapeutic protocol was then changed to trimethoprim-sulfamethoxazole (30 mg/kg, IM, SID for 14 days), resulting in resolution of the infection. According to bacteriological culture of tracheal lavage, both episodes of pneumonia were caused by *Acinetobacter baumannii*.

To identify the etiological agent responsible for the current episode, tracheal lavage samples were collected using a size 8 nasogastric tube for infusion of 65 mL of 0.9% saline solution. Samples were immediately sent to the Animal Microbiological Diagnostic Center (CEDIMA) of Santa Catarina State University (UDESC) after collection. The specimen was plated on 5% sheep blood agar and MacConkey agar and incubated at 37°C for 24 to 48 h. After incubation, pure, hemolytic, translucent, grayish, medium-sized colonies were observed on blood agar, and non-lactose-fermenting colonies on MacConkey agar. Gram staining revealed Gram-negative bacilli. After biochemical tests, the isolate was identified as *Pseudomonas aeruginosa*.

Antimicrobial susceptibility testing was performed using the disk diffusion method according to current Clinical and Laboratory Standards Institute (CLSI) guidelines (1). The isolate was resistant to imipenem, meropenem, aztreonam, ceftazidime, cefepime, ciprofloxacin, enrofloxacin, and ticarcillin/clavulanate. The isolate was susceptible only to aminoglycosides (amikacin). The diameter of the zone of inhibition was interpreted according to human CLSI guidelines (2), with the exception of enrofloxacin, which was extrapolated from veterinary CLSI breakpoints for dogs and cats (3).

**Fig 2:**
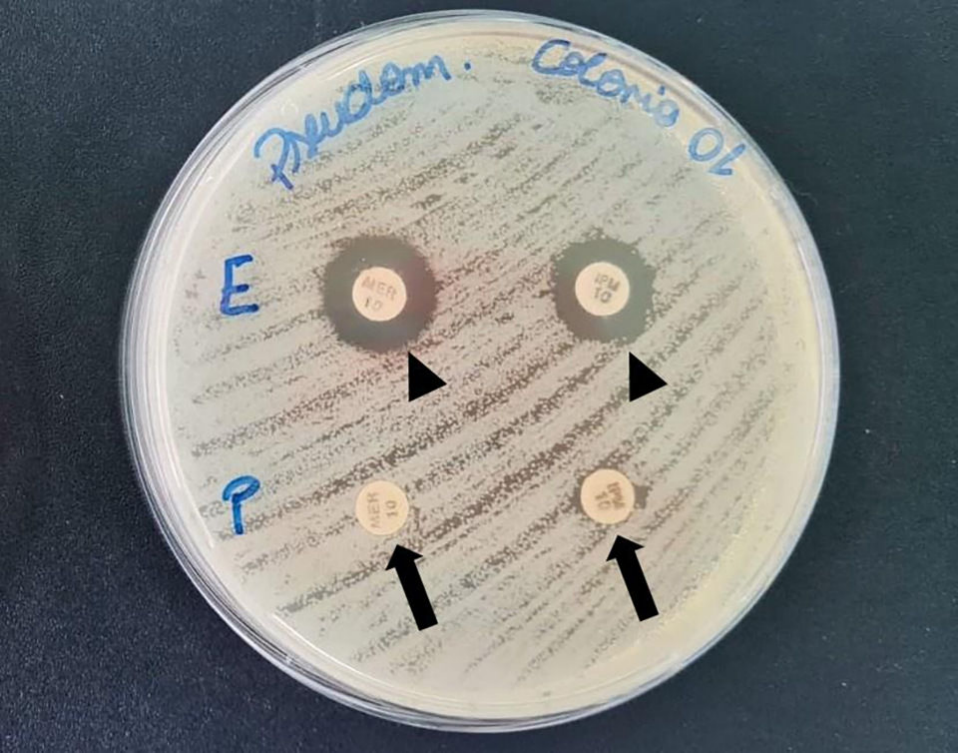
Phenotypic carbapenemase detection in *Pseudomonas aeruginosa*. Black arrow: meropenem and imipenem discs without additives; black arrowhead: discs supplemented with EDTA.

Due to carbapenem resistance, the Inhibitor Test, a phenotypic test for carbapenemase production, was performed following Technical Note no 01/2013 of the Brazilian Health Regulatory Agency (ANVISA, 2013), which indicated the presence of a metallobeta-lactamase enzyme (Fig. 2). A phenotypic test for carbapenemase production was performed using standard disk diffusion with pure imipenem and meropenem disks. In addition, one disk of each antibiotic was soaked with 10 µL of 0.1 M EDTA for 30 min. Plates were incubated at 37°C for 24 h. An increase of ≥5 mm in the inhibition zone around the EDTA-treated disk compared to the corresponding pure antibiotic disk was interpreted as indicative of the presence of an EDTA-chelatable enzyme, suggestive of a metallo-β-lactamase.

After the phenotypic test, the main carbapenemase genes were investigated by polymerase chain reaction (PCR). Screening for the *bla*_IMP_, *bla*_VIM_, and *bla*_KPC_ genes followed Dallenne et al. (4); for the *bla*_SPM_ gene, Sader et al. (5); and for the *bla*_NDM_ gene, Yong et al. (6). Laboratory-developed PCR analysis confirmed the presence of the *bla*_VIM_ gene.

Following sample collection, the animal was treated with trimethoprim-sulfamethoxazole (30 mg/kg, SID) and daily nebulization with saline solution and 20% acetylcysteine due to the initial suspicion of reinfection by *Acinetobacter* (given that the first two episodes were caused by this microorganism). However, before antimicrobial therapy could be adjusted following the isolation of *P. aeruginosa*, the animal’s condition failed to improve with the previously administered trimethoprim-sulfamethoxazole. The clinical status acutely worsened, with signs consistent with sepsis, ultimately leading to death, which precluded the initiation of appropriate antimicrobial treatment.

## DISCUSSION

 The clinical presentation and microbiological findings in this case are consistent with bacterial pneumonia caused by *Pseudomonas aeruginosa* carrying the VIM gene, associated with carbapenem resistance, in a *Boa constrictor occidentalis* previously diagnosed with BIBD. The hypothesis that immunosuppression induced by BIBD predisposes snakes to opportunistic infections is widely recognized and is considered a determining factor in the development of bacterial, fungal, and potential neoplastic processes (7–10).

Bacterial infections in snakes are commonly caused by Gram-negative bacteria due to the opportunistic nature of their microbiota (11–13). When the respiratory system is affected, the bacterial genera most frequently associated include *Aeromonas*, *Pseudomonas*, *Salmonella*, *Klebsiella*, and *Proteus* (11, 14), consistent with the agent identified in the present case.

*Pseudomonas aeruginosa* is a Gram-negative, non-fermenting bacterium with recognized opportunistic capacity, frequently associated with pneumonia in immunocompromised and hospitalized animals (15). These characteristics were observed in our report, in which *P. aeruginosa* presented the following results for the biochemical tests: positive oxidase, non-fermentation of glucose, growth on cetrimide agar with pigment production, negative for the indole and esculin hydrolysis tests, and positive for the urea, malonate, motility, arginate, and citrate tests.

The species possesses a wide array of virulence factors, such as biofilm formation, exotoxin production, and specialized secretion systems, which promote persistence in hospital environments and debilitated hosts (16). These features help explain the therapeutic challenges faced in this case, in which the bacterium demonstrated acquired resistance to multiple antimicrobial classes, despite prior successful treatments.

The adoption of human CLSI standards for interpretation was necessary, as reference values for reptiles are not yet established. The veterinary CLSI was also not used, because most antimicrobials only have reference values for the MIC, and the veterinary CLSI itself recommends extrapolation to the human CLSI when there are no protection zone values for animal isolates. While this limitation may influence the precision of data interpretation, the use of available guidelines provides a consistent framework for comparative purposes and represents the most appropriate alternative until reptile-specific breakpoints become available.

The presence of the VIM gene confers resistance to carbapenems, antimicrobials generally considered last-resort drugs in both human and veterinary medicine (4). The identification of this gene in *P. aeruginosa* isolated from a snake has epidemiological significance, suggesting that reptiles may act as reservoirs of antimicrobial resistance with potential implications for public health. Several hospital outbreaks in humans have been described worldwide due to *P. aeruginosa* carrying the VIM gene (17, 18); however, in animals, descriptions are rare, especially in reptiles.

*P. aeruginosa* has already been reported as one of the predominant bacteria isolated from the oral cavity of snakes of the genus *Python spp*., where 21 of 36 (58.3%) bacterial isolates were resistant to three or more antibiotic classes (19). In another report, *P. aeruginosa* exhibited resistance to multiple antimicrobial groups, including last-resort drugs such as colistin, and carried β-lactam resistance genes such as *bla*TEM, *bla*OXA, and *bla*AmpC (20). Despite these reports, most studies on antimicrobial resistance in snakes have focused on *Salmonella* spp. as carriers of resistant strains (21, 22). Thus, this case broadens the understanding of resistant bacterial pathogens involved in infectious processes in reptiles.

Another relevant aspect is the role of captive management in selective pressure for bacterial resistance. The growing popularity of snakes as companion animals and their use in conservation programs has favored the emergence of more resistant pathogens in these species (23). Repeated and often indiscriminate antimicrobial use in veterinary medicine, frequently without susceptibility testing, is a critical factor in the development of resistance (24–26). In the present case, prior exposure to different classes of antimicrobials, including aminoglycosides and sulfonamides, may have contributed to the selection of multidrug-resistant *P. aeruginosa* strains.

The zoonotic relevance of antimicrobial resistance in reptiles should not be underestimated. Previous studies have shown that *P. aeruginosa* strains can colonize both snakes and their handlers, highlighting the potential for interspecies transmission (27). This reinforces the need to adopt a One Health perspective, recognizing that antimicrobial resistance transcends the boundaries between human and veterinary medicine (28). The identification of a VIM-producing isolate in a captive reptile emphasizes the importance of microbiological surveillance not only in hospital settings but also in breeding facilities and conservation programs.

Furthermore, the unfavorable clinical outcome, despite therapeutic intervention and supportive care, illustrates the limitations of antimicrobial options against multidrug-resistant strains. This underscores the need for rapid diagnostic protocols, including culture, susceptibility testing, and molecular analyses, before defining therapy. Preventive measures, such as proper environmental management, stress reduction, and continuous health monitoring in immunosuppressed animals, also play a crucial role.

In conclusion, this report presents the first description of pneumonia in *Boa constrictor occidentalis* associated with *Pseudomonas aeruginosa* producing a VIM-type metallobeta-lactamase. It highlights the urgent need to better understand the dynamics of antimicrobial resistance in reptiles, considering its clinical, epidemiological, and zoonotic relevance. This evidence supports the implementation of rational antimicrobial use protocols in captive animals and reinforces the importance of integrated strategies within a One Health framework. The report had the consent of the animal’s owner.

## SELF-ASSESSMENT QUESTIONS

1. When performing the phenotypic inhibitor test, which substrate can inhibit metallo-beta-lactamase enzymes?
  - Clavulanic acid
  - Oxacillin
  - Peptones
  - EDTA
2. What are the main microorganisms involved in bacterial pneumonia in snakes?
  - Bacteria of the genera *Streptococcus* and *Staphylococcus*.
  - Gram-positive bacteria.
  - Bacteria of the genus *Enterococcus*.
  - Gram-negative bacteria.
3. Which of the following is a metallo-beta-lactamase enzyme?
  - TEM
  - VIM
  - CTX-M
  - SHV

## ANSWERS TO SELF-ASSESSMENT QUESTIONS

1. Which substrate prevents the action of metallobeta-lactamase in the phenotypic test?
  - Clavulanic acid
  - Oxacillin
  - Peptones
  - EDTA

Answer: d. Metallobeta-lactamases are enzymes characterized by a binuclear zinc catalytic center and, therefore, are inhibited by ionic chelators, such as EDTA, but are not blocked by beta-lactamase inhibitors that have serine in their active site (e.g., clavulanic acid).

- What are the main microorganisms involved in bacterial pneumonia in snakes?
  - Bacteria of the genera *Streptococcus* and *Staphylococcus*.
  - Gram-positive bacteria.
  - Bacteria of the genus *Enterococcus*.
  - Gram-negative bacteria.

Answer: d. Bacterial pneumonia in snakes is most frequently associated with Gram-negative microorganisms such as *Aeromonas*, *Pseudomonas*, *Salmonella*, *Klebsiella*, and *Proteus*. These bacteria are common commensals in snakes, but under stress or immunosuppression, their opportunistic nature predisposes to respiratory infections.

- Which gene is associated with metallobeta-lactamase enzymes?
  - TEM
  - VIM
  - CTX-M
  - SHV

Answer: b. The Verona integron-mediated metallo-beta-lactamase (VIM) enzyme, encoded by VIM genes, belongs to the class of metallobeta-lactamases and was named after being first isolated in 1999 in Verona, Italy. The first strain was identified in *Pseudomonas aeruginosa*.

**TAKE-HOME POINTS**

- Previous antimicrobial exposure in this Boa constrictor occidentalis may have contributed to the emergence of a multidrug-resistant *Pseudomonas aeruginosa* infection, highlighting the importance of judicious antibiotic use and routine susceptibility testing in reptiles.
- This is the first report of a VIM-producing *P. aeruginosa* in a Boa constrictor occidentalis, underscoring the importance of antimicrobial resistance surveillance in reptiles within a One Health perspective.
- Despite antimicrobial treatment, the infection progressed rapidly to sepsis and death. This emphasizes the therapeutic challenges posed by multidrug-resistant bacteria and the urgent need for rapid diagnostics and targeted antimicrobial protocols in reptile medicine.
- There are currently no breakpoints specifically established for reptiles, which poses a significant challenge for veterinarians when interpreting antimicrobial susceptibility results and selecting appropriate therapeutic options.
